# DataGauge: A Practical Process for Systematically Designing and Implementing Quality Assessments of Repurposed Clinical Data

**DOI:** 10.5334/egems.286

**Published:** 2019-07-25

**Authors:** Jose-Franck Diaz-Garelli, Elmer V. Bernstam, MinJae Lee, Kevin O. Hwang, Mohammad H. Rahbar, Todd R. Johnson

**Affiliations:** 1 Clinical and Translational Science Institute, Wake Forest School of Medicine, US; 2 School of Biomedical Informatics, The University of Texas Health Science Center at Houston, US; 3 McGovern Medical School, The University of Texas Health Science Center at Houston, US

**Keywords:** Clinical data quality, secondary use of clinical data, data quality assessment, model-driven development, clinical and translational science

## Abstract

The well-known hazards of repurposing data make Data Quality (DQ) assessment a vital step towards ensuring valid results regardless of analytical methods. However, there is no systematic process to implement DQ assessments for secondary uses of clinical data. This paper presents DataGauge, a systematic process for designing and implementing DQ assessments to evaluate repurposed data for a specific secondary use. DataGauge is composed of five steps: (1) Define information needs, (2) Develop a formal Data Needs Model (DNM), (3) Use the DNM and DQ theory to develop goal-specific DQ assessment requirements, (4) Extract DNM-specified data, and (5) Evaluate according to DQ requirements. DataGauge’s main contribution is integrating general DQ theory and DQ assessment methods into a systematic process. This process supports the integration and practical implementation of existing Electronic Health Record-specific DQ assessment guidelines. DataGauge also provides an initial theory-based guidance framework that ties the DNM to DQ testing methods for each DQ dimension to aid the design of DQ assessments. This framework can be augmented with existing DQ guidelines to enable systematic assessment. DataGauge sets the stage for future systematic DQ assessment research by defining an assessment process, capable of adapting to a broad range of clinical datasets and secondary uses. Defining DataGauge sets the stage for new research directions such as DQ theory integration, DQ requirements portability research, DQ assessment tool development and DQ assessment tool usability.

## 1. Introduction

There is growing interest in the reuse of routinely-collected clinical data for comparative effectiveness research, patient-centered outcomes research and clinical quality improvement [1]. However, analysis of raw clinical data can yield misleading results [1,2,3]. Data flaws such as inaccuracies and incompleteness are often cited as the cause for this hazard [4,5,6], but a fundamental problem is reuse of clinical data for purposes other than originally intended; usually clinical care and health care administration [2,7,8]. Thus, evaluating the data’s fitness for a specific secondary purpose is crucial to ensure valid analytical results [9,10]. Such evaluation is called a data quality (DQ) assessment [11,12,13].

Current repurposed clinical DQ assessment methodologies found in the literature fall short in supporting design and implementation in two distinct ways. On one hand, tool-driven methods and focus on directly detecting data flaws such as inaccuracies rather than supporting assessment design to evaluate ‘fitness for purpose’ [9,10]. These methods fail to enable systematic assessments by failing to provide a fixed, reproducible sequence of steps. They also tend to be rigid detection algorithms rather than generalizable methodologies [10]. On the other hand, DQ guidelines and theory-based methods define approaches to assess data adherence to more abstract DQ concepts [14,15]. Though these methods achieve much higher potential for broad applicability and generalizability, they usually fail to provide explicit implementation guidance to design and execute DQ assessments [10,15]. Due to their higher level of abstraction, they tend be perceived by users as lacking in clarity, difficult to operationalize, and tedious to implement, although this may be the only available way to conduct systematic, reliable DQ assessments [14].

Model-driven software development and QA, is a well-established branch of software engineering that has enabled the systematic evaluation of software products with a fitness-for-purpose approach [16,17,18,19,20,21]. Based on standard methodologies from this field, we developed the DataGauge framework for the systematic DQ assessment of repurposed clinical datasets. DataGauge satisfies most desiderata for DQ assessment guidelines [10,14] by (1) defining a broadly applicable, systematic and explicit assessment pipeline, (2) being able to cover any repurposed clinical dataset and secondary purpose, (3) fully engaging clinical data reuse teams in DQ assessment design, and (4) being independent of a gold standard. DataGauge integrates disparate tools and techniques into a cohesive process blueprint that aims to standardize the design and implementation of data quality assessments. This work integrates existing DQ assessment methodology techniques and provides a clear functional context for their development. We present our framework in three sections. First, we discuss the theoretical foundation and related work that led to the definition of DataGauge. Then, we define DataGauge in detail, illustrating the procedure with a practical and applied example. Lastly, we discuss DataGauge’s ability to integrate past DQ work in the field, its contributions and future research work that would further enable the reliable reuse of clinical data through the development of DQ assessment pipelines.

## 2. Background

Quality assessment (QA) methods are used in many disciplines other than biomedical informatics. Some of these methods address one or more of the aforementioned limitations but have not yet been adapted to assessment of repurposed clinical DQ. *Basic QA methods* rely on qualitative evaluations (e.g., satisfaction surveys) that provide measures of perceived quality [22]. This type of assessment is usually purpose-driven and based on a general set of guidelines [23] to ensure validity. However, such approaches tend to produce ad-hoc evaluations rather than systematic assessments. To facilitate systematic QA, standards organizations such as the International Organization for Standardization (ISO) have defined *quality control standards* [24] and methodologies [25,26,27,28] that require the definition of quantitative requirements and a systematic approach to test compliance with these requirements. One particularly relevant research field that resulted from the creation of these quality control standards is *model-driven software engineering and QA* [29]. This field focuses on developing methods to support the explicit definition of formal requirements and automatic evaluation against these formal requirements. Model-driven QA has not yet been adapted to assess the quality of repurposed clinical data.

Model-driven software development and QA is a well-established branch of software engineering that has reduced the number of errors in complex software (i.e., improved software quality) [16,17,18,19,20,21]. The similarities between DQ and software quality suggest that these methods can be adapted to assess repurposed clinical DQ. The adaptation of these methods is likely to be viable for two reasons: (1) data can be evaluated for quality just like any other product [30] and (2) model-driven data validation has been successfully done on non-clinical administrative data [31]. At the highest level these model-driven software QA methods share three stages: (1) Evaluation of needs and scope definition [16,32,33], followed by (2) Explicit modeling of product specifications (i.e., the quality requirements) based on the needs [16,31,32,33,34], and (3) Evaluation of the product based on the previously-defined requirements [16,31,32,33,35]. Though these stages do not explicitly define the criteria and requirements needed for assessment, they structure the assessment process to make it systematic. This process can be adapted to the secondary use of clinical data as follows: (1) conduct a data needs and scope assessment in the context of the analysis question, (2) develop specifications, which includes the explicit definition of the data needs in a model as well as the definition of DQ requirements and, (3) assess the data according to the DQ requirements. We applied this three-stage approach to our clinical data reuse and extraction requests submitted to our local clinical data warehouse and data team for several different use cases. By iteratively working through this process with each use case we were able to define a concrete pipeline for the development and execution of systematic DQ assessment for repurposed clinical data. We describe this process in the next section along with a concrete application example.

## 3. The DataGauge Process Overview and Example

**Figure 1:**
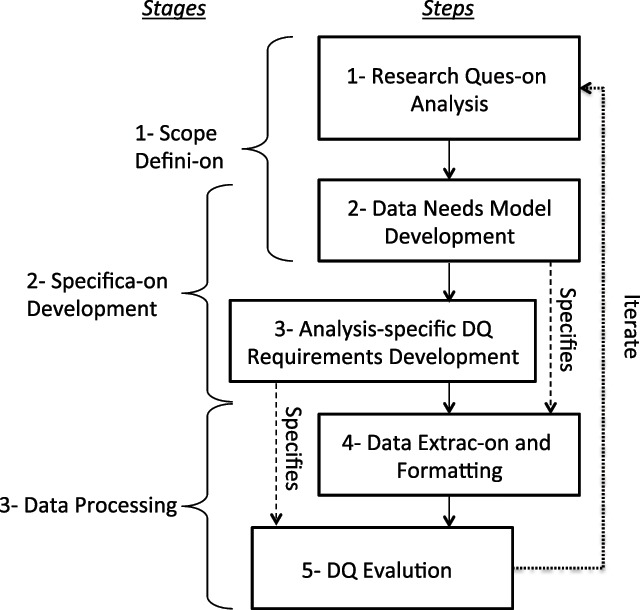
DataGauge, an iterative analysis-specific DQ assessment method for the secondary use of clinical data. This process defines the general stages and steps for analysis-specific DQ assessment using data models and an analysis-specific DQ standard.

DataGauge proposes that the three stages of QA be completed by iteratively executing five concrete steps (see Figure 1): (1) Define information needs based on the analysis question and analytical methods, (2) Develop a data needs model (DNM) that formalizes the data needs, (3) Develop analysis-specific DQ requirements based on the analytical purpose, the DNM and the dimensions of DQ, (4) Extract data from the source data set to fit the DNM, and (5) Evaluate the extract according to the DQ requirements and flag all data that infringe on the DQ assessment standard. These flags can then be used to clean the dataset. Note that DataGauge is defined at a high level of abstraction because it aims to accommodate any kind of clinical data and secondary purpose. This is necessary given the broad range of possible secondary purposes as well as the qualitative nature of DQ. However, DataGauge defines specific and concrete steps that fully define a pipeline for DQ assessment design and execution. DataGauge is also designed to accommodate current and future DQ guidelines through the definition of explicitly defined DQ requirements dependent on the application and assessed dataset.

In the description below, we provide a concrete example as an illustration of each step of the process and preliminary viability validation. We used DataGauge to assess DQ for a repurposed clinical dataset and address the challenges of analysis-specific DQ assessment. The analytical purpose was to determine whether prednisone, a commonly-prescribed corticosteroid, is associated with weight gain. We chose this association because weight gain is a known and clinically-significant side effect of prednisone [36] that is likely to be detectable through retrospective review of clinical data. Our data source was a CDW containing routinely recorded clinical data from six academic outpatient clinics in a large metropolitan area in the southern United States.

### 3.1. Scope Definition Stage

The DataGauge process begins by having a domain expert and statistician *explicitly define an analytical approach to address the research question, along with the information needed to answer the research question*. This is common in statistical analyses [37] and helps to achieve consensus regarding DQ assessment scope. This is not always required in other DQ assessment methods, leaving vagueness in the DQ assessment scope [11,31,38,39,40,41]. For our example, any analysis would require information about the patients, their prednisone exposure, and their weight over time.

**Figure 2:**
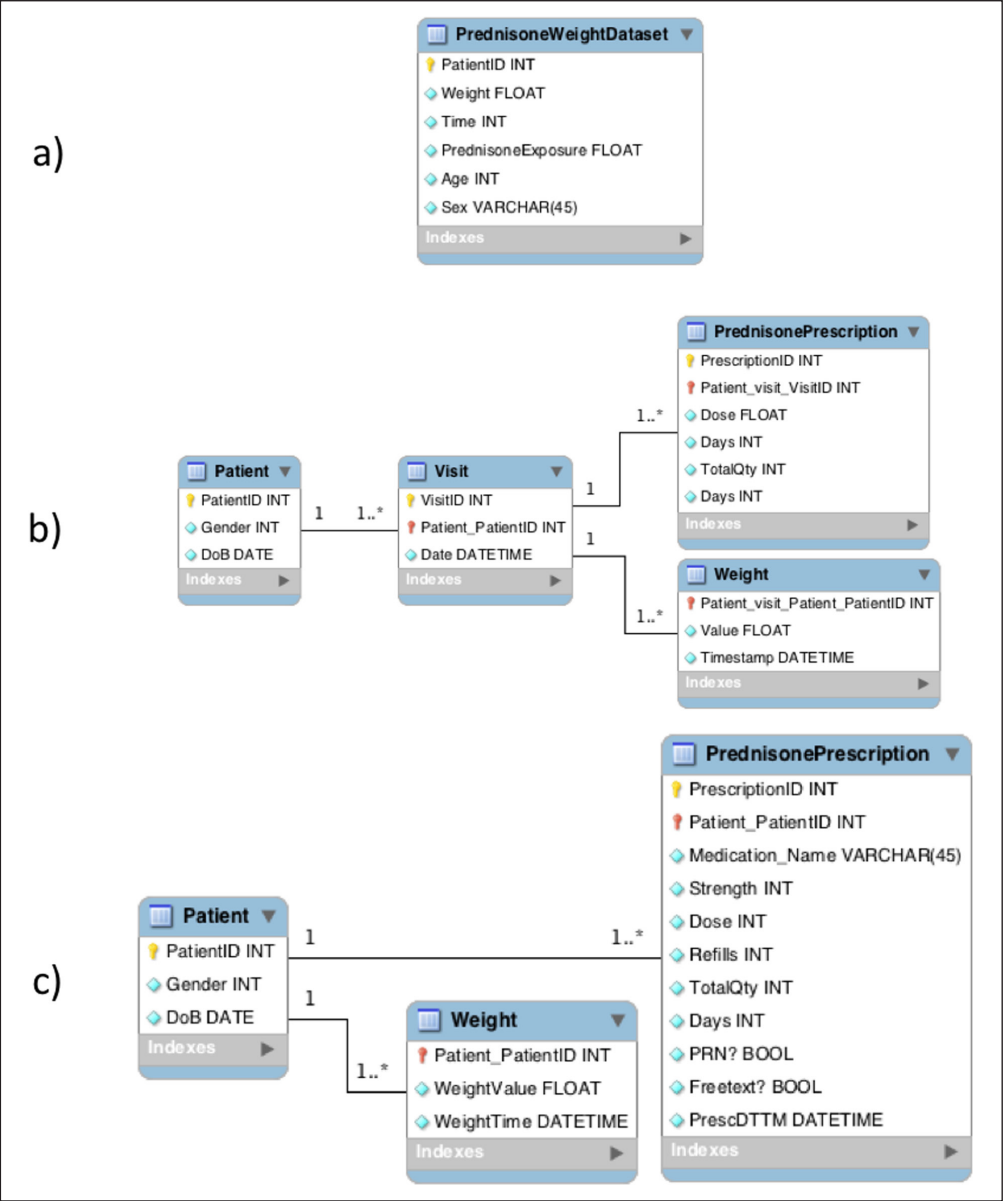
Evolution of the data needs model for the purpose of assessing a relationship between prednisone and weight gain using repurposed clinical data. This data model defines the data needs for the evaluation of an association between prednisone and weight gain. **a)**, **b)** and **c)** show the three versions of the DNM; one for each iteration. Note how the first DNM (a) obscures the observations of interest and their relationships, whereas the third (c) makes these explicit and makes it possible to specify cardinality requirements among them.

*Second, DataGauge requires the analytical team to develop a formal and explicit DNM* from the information needs defined in the previous step. We suggest the use of Unified Modelling language-based (UML) entity-relationship diagrams [42,43] for the definition of these models. The DNM explicitly and unambiguously defines the ideal analytical dataset, a step often overlooked in other DQ assessment methods [11,38,40,41,44,45]. The model serves as a design specification document that defines variables and their relationships but also the scope based on analytical requirements. The qualities of a satisfactory DNM are difficult to define generically because they heavily depend on the analytical purpose. However, we encourage DNM designs to be in at least third normal form [46] or, equivalently, follow a tidy data format [47] to ensure repeatable data structures design, promote systematicity and simplify downstream extract-transform-load and DQ assessment tasks. For our example, we used a UML-based database modeling tool (MySQL workbench data modeler; Oracle Corp., Redwood Shores, CA) to develop the DNM. A team composed of a clinician, a statistician and an informatician (the first author), who also played the role of database administrator, developed the final UML diagram for the research question (Figure 2c). The team created a series of models and discussed their ability to satisfy the analytical purpose as well as data availability in the CDW based on the information needs defined in the previous step (i.e., Patient demographics, prednisone exposure and weight over time). Figure 2 shows the iterative improvement of the DNM from (a) a single-table format that is not tidy data-compliant into (b) a tidy data compliant model with four observational units (i.e., Patient, Visit, Prednisone Prescription and Weight). The final DNM (c) improves on the tidy-data compliant model by removing the Visit observational unit, which is not directly relevant to the research question. The final model is also adapted to fit the data available in the CDW (e.g., changes in the variables describing the prednisone prescription).

### 3.2. Specification Development Stage

*The third step is to define an analysis-specific DQ standard composed of DQ requirements* that fully describe a fit-for-purpose dataset based on the DNM. The DNM serves as an overview and map of the analytical dataset. In this step, it enables the research team to ensure coverage of every value, variable, observation, table and dataset. We define DQ requirements as explicitly-defined constraints that describe the testable features that a dataset must meet to support answering the research question. This step is specific to the research question and DNM (i.e., the dataset and its intended purpose). The DQ standard allows the analytics team to explicitly define and agree on DQ requirements for a particular case. We suggest the use of Object Constraint Language (OCL) paired to the entity-relationship diagrams suggested in step 2 as a possible implementation route [48,49,50,51]. Specifying DQ requirements (i.e., DataGauge Step 3) is a complex, but crucial, task because the analyses rely on requirements to identify possible DQ issues. Although this is a complex task because it requires integrating multiple information sources (i.e., research question, data model and DQ theory knowledge) it has historically been left to domain experts for ad hoc development with no concrete guidance [41,44,45]. This is the *main source of ambiguity in DQ assessment methods as well as a major threat to systematicity. Specifications development has been the target of much recent DQ research* [14,15,52,53]. As new guidelines resulting from this research are developed, they can be integrated into DataGauge.

DataGauge, provides specific, structured guidance to address the complexity of the DQ requirement definition task by integrating existing DQ work outside the clinical data reuse literature. We use two dimensions to guide the definition of requirements: (1) formal levels of data granularity [54] to support thorough evaluation of the DNM and the thorough definition of DQ requirements and (2) existing DQ dimensions and approaches for assessing them [15,39,55,56]. DataGauge combines these dimensions to ensure comprehensive coverage of DQ issues.

For our example, DQ requirements were defined in the form of Boolean expressions and Object Constraint Language (OCL) constraints [49]. We chose OCL due to its integration with the UML diagrams previously used for the data models [48,51,50]. We generated DQ requirements iteratively and collaboratively. We considered the DNM at different levels of data granularity (e.g., single value, multiple values, observation, observational unit, dataset, etc.) [57] in light of each DQ dimension then tied them to a specific DQ assessment approach as integrated in Table 1. For example, when we combined the accuracy dimension with the single value level for the final DNM within the medication table, we generated requirements such as “Dose must be positive” or “Refills must be positive or 0”; both of these requirements were mapped to a range checking method. The concordance dimension at the observation level yielded criteria such as “the prescription date should be later than the patient’s date of birth” which was mapped to the semantic profiling DQ check method. At the observational unit or table level we assessed the timeliness of the data with the “Patient has a second weight measurement within 4 months of the first prescription” requirement. This requirement was also mapped to the semantic profiling check method. The process of running through each DQ dimension and DNM subset was repeated until the analytics team deemed the DQ standard adequate. A sample of the resulting requirements is shown in Table 2.

**Table 1:** DQ requirement development guidance table. Integrated and modified from Wang & Strong’s classification of data quality dimensions (1996) [39] and Borek et al.’s classification of data quality assessment methods (2011) [44].

	Data Quality Dimensions				

Data Granularity Levels	Correctness and Plausibility	Completeness	Concordance	Representation	Timeliness

*Cell/Value*	Domain analysis, Data Validation, Lexical analysis	Domain Analysis, Lexical Analysis	Domain Analysis	Column Analysis, Lexical Analysis, Schema Matching	Domain Analysis
*Column/Variable*	Column Analysis, Data Validation, Semantic Profiling	Column Analysis, Domain Analysis	Column Analysis, Data Validation	Column Analysis, Schema Matching	Column Analysis, Domain Analysis
*Row/Observation*	Domain Analysis, Semantic Profiling	Domain Analysis, Semantic Profiling	Domain Analysis, Semantic Profiling	Domain Analysis, Schema Matching	Domain Analysis, Semantic Profiling
*Table/Observational unit*	Domain Analysis	Domain Analysis, Column Analysis	Column Analysis, Semantic Profiling	Schema Matching	Semantic Profiling, Domain Analysis
*Multiple Tables/Dataset*	Semantic Profiling, PK/FK analysis, Column Analysis	Domain Analysis, Semantic Profiling	Domain Analysis, PK/FK Analysis, Semantic Profiling	Column analysis, PK/FK Analysis, Semantic Profiling, Schema Matching	Semantic Profiling, Domain Analysis
*Multiple Databases/Multiple Datasets*	Semantic Profiling, Domain Analysis, Column Analysis	Domain Analysis, Semantic Profiling	Semantic Profiling, Domain Analysis	Column analysis, Schema Matching, Semantic Profiling	Semantic Profiling, Domain Analysis

### 3.3. Data Processing Stage

The last two steps implement the specifications developed in steps 2 and 3, beginning with the *fourth step: extraction and formatting of the repurposed dataset* from its database of origin. The DNM guides data extraction from the original database. The database administrator creates a schema with tables matching the DNM then loads the source clinical data into the tables. This schema should have all database rules such as variable type definitions, primary key rules, table relationship rules and other data validation triggers built in. In other words, the DNM serves as a dataset blueprint for extraction. Using this predefined schema to load the extracted data ensures that values match the agreed upon data model, variable types and database relationships. This step is an initial representational DQ test; if the data are not in the right format or variable types do not match, the database software should produce an error. For our example, the final DNM served as a data specification document to guide data extraction. Database tables were created to match the DNM and the raw data were extracted from the source database into the DNM schema using standard SQL queries.

The *fifth and last step consists of evaluating DQ based on the previously defined DQ requirements*. Appropriate DQ test methods [58] are implemented to test each DQ requirement. This process evaluates DQ requirements compliance and flags discrepancies. Note that if the DQ requirements in a standardized, machine-readable way, this step can be easily automated. The resulting flags allow further analysis, data diagnosis and imputation [59]. Several indicators (i.e., DQ measures) can be calculated from these flags as measures of DQ (e.g., compliance percentage for each variable or patients with no data flaws divided by the total number of patients). These results provide quantitative evidence of non-compliant data and can serve as a basis for experts to judge their DQ (i.e. their fitness for purpose). At this point, a visual representation of the DNM can be used to organize, present and interpret the DQ assessment results. In our example case, we evaluated the quality of extracted data based on the third version of the DQ standard (Figure 2c). We covered analysis-specific DQ requirements as well as generic requirements to test accuracy and believability of the data. Of 52 requirements, 17 were analysis-specific. Analysis-specific requirements tended to be more complex and involved a larger number of variables. Table 2 shows how the requirements evolved over iterations; note the increasing precision and analysis-specificity (e.g., “2 values per patientID” in iteration 2 followed by “50 percent patients with 2 weight measures within 4 months of first prescription”). Each new DNM represented a specific data model designed to satisfy the same analytical purpose; each iteration for the DQ requirement created an increasingly complete, refined and analysis-specific set of requirements.

**Table 2:** DQ requirement examples as they were generated with their respective percentage of compliance. The requirements became more specific and analysis-specific with each iteration.

Iteration	DQ Dimension	Variable Granularity	Variable(s)	Analysis Specific	Requirement	DQ assessment method	DQ Result (% Compliance or Pass/Fail)

1	Accuracy	Value	Gender	No	In {‘M’,‘F’,‘U’}	Data Validation	99.99
	Accuracy	Value	WeightValue	No	>0	Range Checking	92.65
	Believability	Value	WeightValue	No	<400	Range Checking	99.95
	Accuracy	Value	Strength	No	>0	Range Checking	97.37
	Believability	Value	Strength	No	<2* [Max dose]	Domain Analysis	100
	Accuracy	Value	Dose	No	>0	Range Checking	51.68
	Believability	Value	Dose	No	<2* [Max pills at min strength]	Domain Analysis	100
	Accuracy	Value	Refills	No	>=0	Range Checking	100
2	Accuracy	Value	WeightTime	No	>[System Installation Date]	Data Validation	100
	Accuracy	Column	PatientID	No	Unique	Column Analysis	100
	Concordance	Row	WeightTime, DoB	No	Timestamp > DoB	Domain Analysis	100
	Concordance	Row	PrescDTTM, DoB	No	PrescDTTM > DoB	Domain Analysis	100
	Concordance	Table	PatientID, WeightTime, WeightValue	Yes	Patient weights on prescription date are less than 2% apart	Domain Analysis	92.45
	Completeness	Table	PatientID, WeightValue	Yes	2 weight measurements per patient	Domain Analysis	85.92
	Completeness	Line	PatientID, WeightTime	Yes	Patient has weight measurement on prescription date	Domain Analysis	97.54
	Timeliness	Table	PatientID, WeightTime	Yes	Patient has second weight measure within 4 months of prescription	Domain Analysis	48.62
3	Amount of data	Table	Strength, Dose, Days, Refills	Yes	Can calculate total milligrams prescribed for 50% of prescriptions	Domain Analysis	Failed
	Amount of data	Table	Patient, PRN	Yes	Less than 25% PRN prescriptions	Domain Analysis	Passed
	Amount of data	Dataset	PatientID, WeightTime	Yes	50% patients 2 weight measures within 4 months of first prescription	Domain Analysis	Failed
	Completeness	Dataset	PatientID, WeightValue, WeightTime, PrescriptionTable	Yes	Patients with at least 2 unflawed weights after an unflawed prescription	Domain Analysis	13.1
	All	Dataset	All Variables	No	Patient records with no general DQ flaw	Domain Analysis	2.93

Overall DQ tests revealed several DQ flaws in our test case (Table 2). We were able to identify specific DQ issues such as inaccuracies (e.g., 84 weight values were above 400 kg), inconsistencies (e.g., 56 instances where weight changed more than 20 percent over 2 days) and incompleteness (e.g., 43,135 patients with less than two weight measurements within 3 months of the prescription). This showed the approach’s effectiveness at catching DQ issues and screening data at the basic data level. We also excluded 14.1 percent of the patient records as they contained a single weight measurement and weight gain can only be calculated with two or more. We flagged all data items that violated DQ criteria and then calculated the number of patients with no flagged data in their records, having at least two weight measurements after their first prednisone prescription. Thus, only 2,379 patients out of 80,990 (13.1 percent) could be used reliably for analysis without any further data quality assessment, cleaning, imputation or manual chart review. This raised questions about the reliability of direct analysis and potential conclusions drawn from such analysis.

## 4. Discussion

DataGauge specifies a procedure that supports the systematic design and implementation of clinical dataset and secondary purpose-specific DQ assessments. DataGauge differs from previous work by combining guideline [14,15] and DQ test [41,44] approaches to DQ assessment into one process, integrating clinical data extraction [60] and assessment. DataGauge contributes to the field of clinical data reuse for comparative effectiveness, patient-centered outcomes research and quality improvement in two ways: (1) it provides an explicit implementation method for the variety of guidelines available for DQ assessment and (2) it describes a new methodological layer to (at least partially) satisfy the four requirements of “fitness for purpose” DQ assessment methodologies [14,56]. DataGauge is systematic because it defines an explicit set of steps to prevent the ad-hoc application of DQ tests with no “fitness for purpose” testing plan. Being a direct adaptation of software QA methodologies [16,32,61], it is also domain expert knowledge-based. In addition, it builds upon Kahn et al.’s framework for pragmatic DQ assessment [11] adapting its single-site DQ assessment fitness evaluation. DataGauge accounts for the task-dependent nature of DQ by dedicating three out of five steps to the design of the dataset and purpose specific documents (i.e., the DNM and DQ requirements). DataGauge also engages users by enabling communication, discussion and iterative design as a basis for DQ assessment implementation. It is designed for collaborative execution by a team of domain experts (e.g., clinicians), data users (e.g., researchers, clinical administration, etc.), informaticians, statisticians, and database administrators. Finally, DataGauge is independent from the availability of gold-standard data, as it leverages expert knowledge to explicitly define a purpose-specific standard.

Recent work in the field of DQ for EHR data reuse has also aimed at enabling systematic assessments in two ways. On one hand, other DQ assessment processes have been published [62,63] but they do not provide a methodology that is systematic yet purpose-specific. For example, Reimer et al. describe a six-step process to assess clinical data based on the dimensions of DQ, focusing on issues such as patient matching across databases and evaluating record completeness rather than testing fitness for a specific purpose. On the other hand, the field has focused on establishing the theoretical basis for DQ assessment as well as assessment guidelines. For example, DQ reporting guidelines help structure the definition of DQ assessments [15], development of a harmonized terminology to facilitate discussion of DQ assessments [55], the development of an ontology-based DQ assessment framework [64] have all set a strong theoretical basis for systematic DQ assessment method development. One of the most impactful contributions has been the 3 × 3 DQ assessment guideline based on the literature, EHR data assessment and expert review that provides clinical data analysts with a clear framework to test specific aspects of DQ in a purpose-specific way [9]. However, the existing literature does not provide much guidance on systematic DQ assessment design and implementation processes.

DataGauge defines a series of concrete steps for the design and execution of DQ assessments to support implementation and relies on the definition of explicit DQ requirements (Step 3). However, DQ requirement definition is a complex, cognitively taxing activity. The complexity stems from the need for mixed quantitative-qualitative reasoning as well as information from multiple data sources such as the intended analytical design or purpose [9,15], data source descriptions [40,41], data management constraints [44,57] and DQ theory [14,15,38,55]. DataGauge supports this process by: 1) Providing a systematic process to encode and define and the assessment scope and assessed data in explicit, unambiguous terms through a DNM that integrates knowledge about the purpose-specific data model, the data available in the clinical data source, its clinical data types, 2) Providing preliminary guidance to account for DQ theory as an initial guideline (see Table 1) that integrates general DQ dimensions [39] with levels of data granularity [44,57] and practical granularity-specific DQ testing approaches [44] and 3) Enabling the use of existing DQ assessment guidelines through DQ requirement definition.

Users can enrich the DataGauge process by defining their requirements using existing guidelines that may better serve their specific applications. For example, we could apply the 3 × 3 DQA guidelines [14], a recently published guidance framework for the design of systematic DQ assessments, to our illustrative example (see Figure 1c and Table 2). Data reuse teams can develop DQ requirements to ensure fitness for purpose for each dimension of 3 × 3 DQA for at all data elements in the DNM, at all levels of data granularity to make this process systematic, comprehensive and potentially repeatable. Using our example, we would decompose the DNM into multiple tables, variables, observations (e.g., weight and associated timestamp), etc. Then we would use each model subset and examine it in light of each DQ Dimension-Construct combination. For example, when we combine the “Correct” dimension with the “Variable” construct at the single value level of granularity for the final DNM we may generate requirements such as “Dose must be positive” or “Refills must be positive or 0”. Both requirements can be mapped to a range checking method (Table 1). This methodical dataset decomposition approach paired to the guidance provided by the 3 × 3 DQA framework would enable data reuse teams to ensure systematic DQ requirement definition.

### 4.1. Limitations

DataGauge has limitations that stem mostly from the vast number of ways each step may be executed. This is an artifact of the general problem of *defining a procedure to assess the quality of data or “fitness for purpose”*. This is also the case for many DQ frameworks, guidelines and methods and, in the case of DataGauge, it has multiple repercussions. First, the definition of specific DQ requirements for each possible DQ threat is human resource intensive. However, the threats posed by data repurposing [2,8] demand such a thorough evaluation. DataGauge also supports the explicit definition of these features, enabling clearer and more transparent communication [15,55] of DQ assessment designs, requirements and results. Using DQ requirements rather than testing tools also opens new research directions such as defining methods for DQ requirement portability. DataGauge does not define any specific standard to encode these requirements, limiting the potential reusability of DQ requirement development work; further research is needed in this area. A promising avenue to enable the portability of DQ requirements is archiving. For example, DQ requirements could be archived based on the clinical data types (e.g., demographics, labs, medications) that they touch on and the purpose for which they were developed (e.g., disease prevalence estimation). This archive would enable previously-defined DQ requirement retrieval for future studies involving the same clinical data types and purpose. This would progressively reduce the human-intensive nature of DataGauge but further research and development is needed to make such systems available. Second, DataGauge does not provide guidance on the number of iterations needed to ensure reliable evaluation of fitness for purpose (i.e., knowing for certain when all requirements have been defined). However, this may not be definable because of wide variability in secondary purposes. DataGauge structures the DQ assessment process rather than defining an algorithm. DataGauge, in its current form, cannot be automated because it requires purpose-specific domain knowledge and relies in part on human judgment [14]. Nevertheless, DataGauge is a systematic process that can support future automation efforts and the systematic implementation of published DQ assessment guidelines.

### 4.2. Future Work

Defining the DataGauge process opens many research avenues that require the involvement of the broad community of researchers making secondary use of clinical data. Beyond additional empirical validation of DataGauge’s effectiveness at guiding clinical data reuse efforts [65], future research should address practical barriers to DQ assessment implementation. We propose further development and integration of the DataGauge process-oriented approach to DQ assessment. Some development avenues to pursue are the development of automated DQ requirement generation from data model and metadata, the integration of harmonized DQ terminology within implementation process [55] and the development of an interactive tool for DQ requirement development capable of automated evaluation following the DataGauge process. Some integration research ideas are the implementation of an interactive version of the DQA 3 × 3 guideline [14] for the generation of DQ requirements within an interactive DataGauge process platform, the integration of the DataGauge process with existing DQ assessment tools [66] and facilitating the formal definition of DQ tests by integrating Johnson et al.’s DQ ontology and DQ measurement definitions [64,67].

We also propose pursuing research to build DQ assessment practices into the learning health care system along three research directions: DQ infrastructure development, DQ portability [68] and DQ usability [69]. Infrastructure could be developed by researching the use of formal, machine readable models to automate the extraction of clinical data directly from DNMs and the development of DQ requirement databases based on clinical data types, domain knowledge and intended secondary purpose to be reused for automated DQ requirement generation or suggestion. To enable DQ portability, we propose the definition of a formal, machine-readable DQ requirement encoding standard [70] and testing the viability of DQ requirement portability across repurposed clinical datasets and secondary purposes. DQ requirement warehousing and cataloguing methods should also be investigated to enable intelligent retrieval for reuse based on clinical data types and secondary use purpose. An accessible library of DQ requirements containing previously-defined requirements would enable reducing the number of man-hours to complete a thorough DQ assessment. Finally, DQ usability research should explore the cognitive challenges DQ requirement generation process using think aloud sessions with data scientists designing DQ assessments and verify the usefulness of explicit DQ assessment design documents (i.e., DNMs and DQ requirement sets) to improve DQ assessment.

### 4.3. Conclusion

We have presented DataGauge, a model-driven, iterative, team-based process to carry out purpose-specific DQ assessment for the secondary use of clinical data. It addresses a major barrier in the field: the lack of an explicit procedure for the design and implementation of DQ assessments. DataGauge also allows its users to systematically implement their preferred DQ assessment guideline for the definition of DQ requirements. DataGauge has two core contributions: (1) the integration of a number of disparate DQ assessment methods and techniques into a cohesive implementable process and (2) the explicit definition of a DQ assessment design and implementation methodology. This work also contributes by opening new avenues of research on systematic DQ assessment methods such as the development of a platform for shareable DQ requirements and the integrated implementation of existing DQ assessment guidelines.
